# Promoting Safe Injection Practices, Substance Use Reduction, Hepatitis C Testing, and Overdose Prevention Among Syringe Service Program Clients Using a Computer-Tailored Intervention: Pilot Randomized Controlled Trial

**DOI:** 10.2196/19703

**Published:** 2020-09-29

**Authors:** Karli R Hochstatter, Shawnika J Hull, Ajay K Sethi, Marguerite E Burns, Marlon P Mundt, Ryan P Westergaard

**Affiliations:** 1 Columbia University School of Social Work New York, NY United States; 2 Department of Prevention and Community Health Milken Institute School of Public Health The George Washington University Washington DC, DC United States; 3 Department of Population Health Sciences University of Wisconsin School of Medicine and Public Health Madison, WI United States; 4 Department of Family Medicine and Community Health University of Wisconsin School of Medicine and Public Health Madison, WI United States; 5 Department of Medicine University of Wisconsin School of Medicine and Public Health Madison, WI United States

**Keywords:** hepatitis C virus, intravenous drug abuse, drug overdose, harm reduction, web-based intervention

## Abstract

**Background:**

Syringe service programs (SSPs) are safe, highly effective programs for promoting health among people who inject drugs. However, resource limitations prevent the delivery of a full package of prevention services to many clients in need. Computer-tailored interventions may represent a promising approach for providing prevention information to people who inject drugs in resource-constrained settings.

**Objective:**

The aim of this paper is to assess the effect of a computer-tailored behavioral intervention, called Hep-Net, on safe injection practices, substance use reduction, overdose prevention, and hepatitis C virus (HCV) testing among SSP clients.

**Methods:**

Using a social network–based recruitment strategy, we recruited clients of an established SSP in Wisconsin and peers from their social networks. Participants completed a computerized baseline survey and were then randomly assigned to receive the Hep-Net intervention. Components of the intervention included an overall risk synthesis, participants’ selection of a behavioral goal, and an individualized risk reduction exercise. Individuals were followed up 3 months later to assess their behavior change. The effect of Hep-Net on receiving an HCV screening test, undergoing Narcan training, reducing the frequency of drug use, and sharing drug equipment was assessed. The individual’s readiness to change each behavior was also examined.

**Results:**

From 2014 to 2015, a total of 235 people who injected drugs enrolled into the Hep-Net study. Of these, 64.3% (151/235) completed the follow-up survey 3-6 months postenrollment. Compared with the control group, individuals who received the Hep-Net intervention were more likely to undergo HCV testing (odds ratio [OR] 2.23, 95% CI 1.05-4.74; *P*=.04) and receive Narcan training (OR 2.25, 95% CI 0.83-6.06; *P*=.11), and they shared drug equipment less frequently (OR 0.06, 95% CI 0.55-0.65; *P*<.001). Similarly, individuals who received the intervention were more likely to advance in their stage of readiness to change these 3 behaviors. However, intervention participants did not appear to reduce the frequency of drug use or increase their readiness to reduce drug use more than control participants, despite the fact that the majority of the intervention participants selected this as the primary goal to focus on after participation in the baseline survey.

**Conclusions:**

Implementing computer-based risk reduction interventions in SSPs may reduce harms associated with the sharing of injection equipment and prevent overdose deaths; however, brief computerized interventions may not be robust enough to overcome the challenges associated with reducing and ceasing drug use when implemented in settings centered on the delivery of prevention services.

**Trial Registration:**

ClinicalTrials.gov NCT02474043; https://clinicaltrials.gov/ct2/show/NCT02474043

**International Registered Report Identifier (IRRID):**

RR2-10.2196/resprot.4830

## Introduction

### Background

The concurrent and overlapping epidemics of substance abuse, overdose, and hepatitis C virus (HCV) constitute a public health crisis. In the United States, an estimated 1.7 million people are affected by substance use disorders related to prescription opioids and 652,000 people are affected by heroin use disorders [1]. Moreover, approximately 70,237 Americans died from drug overdoses involving prescription opioids or illicit substances in 2017 alone, a four-fold increase since 1999 [2]. Through the sharing of needles and preparation equipment, injection drug use has fueled the spread of infectious diseases and now serves as the primary risk factor for HCV transmission in the United States [3]. From 2004 to 2014, acute HCV increased by 400% among Americans aged 18 to 29 years [4]. During this same period, there was an 817% increase in treatment admissions for injection of prescription opioids and a 603% increase in admissions for heroin injection [4].

These sharp increases in injection drug use and HCV incidence are concentrated in communities with traditionally poor access to prevention services, affecting rural and suburban residents in particular [5,6]. An epidemiologic investigation conducted to understand the factors associated with the tripling of HCV incidence between the periods of 2004 and 2008 and 2009 and 2010 in 6 contiguous rural counties of Wisconsin found that 94% of infected patients had shared hypodermic needles, drug preparation equipment, or drug snorting equipment [7]. Similar outbreaks of HCV among young persons who inject drugs have occurred across many other communities in the United States, including several Appalachian states, Massachusetts, Indiana, and New York State [6,8-11], and several other jurisdictions have been identified as potentially vulnerable communities to similar outbreaks [12].

Syringe service programs (SSPs) are safe, highly effective programs for promoting health among people who inject drugs. By providing access to sterile needles and other drug paraphernalia, these community-based programs have been shown to reduce the transmission of HCV and HIV among drug-using networks [13-18], without increasing the frequency of drug use [19-21]. In addition to distributing comprehensive prevention materials, SSPs often offer a range of services, including referral to substance use disorder treatment programs, education on safe injection practices, overdose prevention, and counseling and testing for HCV, HIV, and other sexually transmitted diseases. Despite the abundant documented benefits of SSPs, resource limitations prevent the delivery of a full package of prevention services to many in need [22-24].

Computer-tailored interventions (CTIs), in which the output of a persuasive technological system is adapted to the individual, are a low-cost strategy for delivering personalized health information that is specific to the unique psychosocial needs of each individual. Persuasive health technology is a growing field that combines medicine, computer science, and psychology to aid and motivate people to adopt behaviors that benefit them while avoiding the harmful ones and is used for both health promotion, prevention and disease management [25-27]. CTIs have become increasingly common for facilitating a wide range of healthy behaviors, including smoking cessation, diet and exercise, and mammography screening [28]. However, the use of CTIs to promote healthy behaviors among people who inject drugs, particularly in prevention-oriented and other community-based settings such as SSPs, remains to be less studied. CTIs may represent a promising approach for providing a personalized risk reduction plan for people who inject drugs in resource-constrained settings.

### Objectives

Recognizing the growing demand for targeted, evidence-based interventions and the existing infrastructure of SSPs, a computer-tailored risk reduction intervention, called Hep-Net, was developed and implemented through a pilot randomized controlled trial (RCT) [29]. The objectives of this study are to evaluate whether incorporating a computerized behavioral intervention into existing prevention services at SSPs can lead to the adoption of healthier behaviors, including safer injection practices, substance use reduction, overdose prevention, and HCV testing.

## Methods

### Study Population and Settings

From 2014 to 2015, a total of 235 people who inject drugs enrolled in the Hep-Net study. Participants were either clients of an established SSP operating in 2 Wisconsin cities, Madison and Milwaukee, or peers recruited from the social networks of these clients. Eligibility criteria included being 18 years of age or older, having injected drugs in the past 30 days, and willingness to provide contact information for the 3-month follow-up.

To conduct outreach among high-risk populations of people who inject drugs and engage even those who do not regularly use prevention services, we used social network–based referrals to recruit the study sample. SSP clients were informed about the study during a routine needle-exchange encounter, invited to participate, screened for eligibility, and, if eligible, provided with the computerized baseline survey. After completing the baseline survey, participants were randomly assigned to receive the intervention, in which the computer immediately delivered the intervention content, or the control, in which the computer session was terminated after completion of the baseline survey. Upon completion of the baseline visit, study participants in both arms received referral coupons and were encouraged to refer eligible peers. Using the contact information provided at baseline, individuals were contacted 3 months later to complete the follow-up assessment. A protocol that describes in detail recruitment methods and intervention content and provides survey instruments and example content images of the Hep-Net intervention has been previously published [29].

### Risk Reduction Intervention

The Hep-Net intervention aims to increase health-promoting behaviors and reduce risky drug use behaviors among people who inject drugs. Hep-Net targets 4 different behavioral domains: (1) undergoing regular HCV screening, (2) taking steps to prevent opioid overdose, (3) using clean works for every injection, and (4) reducing and ultimately ceasing injection drug use. Components of the intervention included an overall risk synthesis, participants’ selection of a behavioral goal (from 1 to 4) and an individualized risk reduction exercise [29]. The risk synthesis had 2 components: first, positively framed feedback messages were delivered, highlighting healthy behaviors reported by participants that can reduce the risk of HCV transmission and opioid overdose, and second, tailored feedback was delivered regarding specific behaviors associated with increased risk reported by the participant. After selecting a behavioral goal, the participants were delivered a series of screens with feedback and educational content tailored to the individual’s reported risk behaviors and their stage of readiness to change. Finally, during the interactive risk reduction exercise, the participants were asked to select 3 to 5 action steps from a list of 10 to 12 suggested steps that they were willing to do in the following 3 months.

Action steps considered during the individualized risk reduction exercise were tailored to the participant’s readiness to change (as assessed at pretest) and are informed by the transtheoretical model (TTM), which conceptualizes behavior change as a continuum and argues that different processes of change are relevant to different stages of readiness [30]. According to the TTM, individuals who are not yet considering behavior change are in the precontemplation stage. In the contemplation stage, individuals may consider change but have not yet taken steps toward behavioral change. Contemplation is followed by preparation, action, and maintenance [31]. The Hep-Net intervention delivered feedback and risk reduction activities that were informed by the individual’s stage of readiness, as described in the RCT’s published protocol [29].

### Data Collection

The baseline survey and the Hep-Net intervention were delivered from August 6, 2014, to April 16, 2015. Baseline information included demographics, drugs of choice, the duration of injection drug use, incarceration history, HCV status, the frequency of sharing needles, syringes and other works, naloxone (Narcan) training status, the frequency of drug use, and readiness to change each of the 4 targeted behaviors. Three-month follow-up surveys were conducted until August 18, 2015, and the same behaviors were assessed as the baseline questionnaire. All surveys were delivered on laptops and designed to last 20 to 30 min.

In addition to the Hep-Net survey data, HCV testing data were collected and stored at SSP sites through the agency’s standard protocol as well as in the Wisconsin Electronic Disease Surveillance System (WEDSS) in the Department of Health Services for those who tested HCV positive. These 2 additional data sources were used to track HCV testing for those who did and did not complete the follow-up survey, allowing us to measure the HCV testing uptake with greater validity. To assess HCV follow-up testing within 6 months of enrollment for all individuals enrolled in the study, we received participant-level data stored in the WEDSS through a secure, encrypted, and Health Insurance Portability and Accountability Act (HIPAA)-compliant data transfer protocol. Furthermore, archived testing records stored at the 2 SSP sites were manually searched to identify individuals who tested HCV negative. Study participants were matched to the WEDSS and SSP data by first and last name and date of birth. The variables collected from these sources include the date of HCV tests and test results.

### Main Outcomes

We assessed 4 behavioral goals:

- *Receive HCV follow-up testing*: Individuals were considered tested for HCV at follow-up if they were not already aware of being HCV positive at the time of Hep-Net enrollment and if any of the following conditions were true: (1) self-reported at follow-up being tested for HCV since their first study visit, (2) underwent HCV testing at the time of the follow-up survey, (3) an archived record of an HCV test existed 6 months postenrollment at the SSP, or (4) any HCV test was reported to the WEDSS 6 months postenrollment.
- *Get trained to administer Narcan*: Individuals were considered trained to administer Narcan if they self-reported undergoing training for naloxone, or Narcan, in the follow-up survey. Those who were already trained at baseline were excluded from this analysis under the assumption that the knowledge and skills gained from such training last indefinitely.
- *Reduce frequency of sharing drug equipment*: To measure the frequency of sharing needles, syringes or other works, individuals were asked how often they shared (1) needles, (2) cottons or filters, and (3) cookers with another person when they used drugs in the past month. Responses were recorded using a slider-bar feature and ranged from 0% of the time they used drugs to 100%. For this analysis, the frequency of sharing drug equipment corresponded to the highest percentage reported among these 3 pieces of equipment. When response data for one or more of these 3 questions were missing, it was assumed that the data were missing at random. These observations were excluded because we could not make an inference about why these data would be missing or hypothesize what these data would have been.
- *Reduce the frequency of drug use*: To measure the frequency of drug use, individuals were asked how often they used heroin; oxycodone, or OxyContin; methamphetamine (crystal or meth); and cocaine or crack in the past month. Four response categories were available, ranging from never to every day. For this analysis, the frequency of drug use corresponded to the highest frequency of use reported among these drug types and was collapsed into 3 categories: (1) never or less than once a week, (2) more than once a week but not every day, or (3) every day.

Understanding that behavior change often occurs through a series of stages as opposed to a single discrete event and that the Hep-Net intervention may not be powerful enough for some individuals to achieve full behavior change over the course of 3 months, we also assessed individuals’ *readiness* to engage in these 4 health-promoting behaviors. At baseline and follow-up, individuals were presented with each of the 4 behavioral goals and asked to select their readiness to change each behavior. The 5 answer choices ranged from *I am not even thinking about this goal* to *I have reached this goal*, which corresponds with the 5 stages outlined by the transtheoretical model: precontemplation, contemplation, preparation, action, and maintenance. Owing to the low number of individuals observed in the contemplation and precontemplation stages, these stages have been combined into a single stage for this analysis.

### Statistical Analysis

Descriptive statistics were calculated for the baseline variables of the entire study population. Characteristics of the intervention group were compared with those of the control group using Pearson chi-square (if expected cell frequencies are >5) or Fisher exact (if expected cell frequencies are ≤5) tests for testing differences in categorical variables and the two-sample unpaired *t* test (if normally distributed) or Wilcoxon rank-sum test (if not normally distributed) for continuous variables. Baseline characteristics were also examined to determine whether there were any characteristics that differed between those who returned for the follow-up survey and those who were lost, using the same statistical tests.

As the Hep-Net risk reduction intervention presented a menu of behavioral goals, providing the opportunity for behavior change beyond the behavior that was selected, analyses compared all intervention participants (as opposed to only those who selected the particular goal) with control participants. Binomial logistic regression was used to assess differences between intervention and control arms for binary outcomes: receiving HCV follow-up testing (yes or no) and undergoing Narcan training (yes or no). As the frequency of sharing drug equipment is a non-normally distributed outcome, we conducted Poisson regression to analyze the difference between the intervention and control arms in sharing equipment. Ordinal logistic regression was used to assess the difference in the frequency of drug use between the intervention and control arms. Ordinal logistic regression was also used to assess differences in intervention and control participants’ readiness to change each behavior. All analyses were conducted using SAS version 9.4 (SAS Institute). Statistical significance was determined at α≤.05, two-sided.

## Results

### Demographics

During the enrollment period, 235 people who injected drugs provided consent and completed the baseline survey. Of these, 109 people were randomly assigned to the intervention group and 126 were assigned to the control arm. The baseline descriptive characteristics are displayed in Table 1. Among the 235 individuals, 180 (76.6%) were male, 138 (58.7%) were White, 211 (89.8%) had at least a high school diploma or GED, 161 (68.5%) were unemployed at the time of enrollment, and 172 (73.2%) had an annual income of less than US $11,500. A total of 44.7% (105/235) individuals used illicit substances every day at baseline. Heroin was the most common substance used in the past 30 days, with 87.2% (205/235) individuals using heroin, followed by cocaine or crack (147/235, 62.6%), oxycodone or OxyContin (103/235, 43.8%), and methamphetamine (19/235, 8.1%). At baseline, 71.1% (167/235) individuals self-reported ever being tested for HCV before Hep-Net study enrollment, of which 19.2% (32/235) reported testing HCV positive.

Of the 235 individuals who completed the baseline survey, 151 (64.3%) completed the follow-up survey 3 to 6 months postenrollment. Individuals who were lost to follow-up were slightly less likely to have health insurance (81.0% vs 90.0%; *P*=.05) and more likely to be homeless (65.1% vs 47.0%; *P*=.008). This finding is expected, considering that homeless populations are often transient [32], which may lead to high study attrition and are less likely to have health insurance [33].

**Table 1:** Baseline demographic characteristics by intervention and control arm (N=235).

Characteristics		Study arm^a^	
		Control (n=126)	Intervention (n=109)
**Gender, n (%)**			
	Male	92 (73.0)	88 (80.7)
	Female	34 (27.0)	21 (19.3)
**Race, n (%)**			
	White	79 (62.7)	59 (54.1)
	Black or African American	32 (25.4)	34 (31.2)
	Other or multiple	15 (11.9)	16 (14.7)
Age (years), median (IQR)		35 (18)	33 (17)
**Education level, n (%)**			
	Less than high school	17 (13.5)	7 (6.4)
	GED^b^, HSED^c^, or high school diploma	61 (48.4)	50 (45.9)
	At least some college	48 (38.1)	52 (47.7)
**Health insurance, n (%)**			
	No	20 (16.0)	11 (10.1)
	Yes	105 (84.0)	98 (89.9)
**Completed follow-up survey, n (%)**			
	No	49 (38.9)	35 (32.1)
	Yes	77 (61.1)	74 (67.9)
**Currently employed, n (%)**			
	No	92 (73.0)	69 (63.9)
	Yes	34 (27.0)	39 (36.1)
**Children at home, n (%)**			
	No	100 (79.4)	88 (80.7)
	Yes	25 (19.8)	20 (18.4)
**Income,** **US $;** **n (%)**			
	None	31 (24.6)	30 (27.5)
	Less than 11,500	62 (49.2)	49 (45.0)
	More than 11,500	28 (22.2)	30 (27.5)
**Homeless in the past year, n (%)**			
	No	58 (46.0)	51 (46.8)
	Yes	68 (54.0)	57 (52.3)
**Incarcerated in the past year, n (%)**			
	No	82 (66.1)	63 (58.9)
	Yes	42 (33.9)	44 (41.1)
**Substance used in past 30 days, n (%)**			
	Heroin^d^	114 (92.7)	91 (84.3)
	OxyContin or Oxycodone^d^	62 (50.4)	41 (37.6)
	Methamphetamine	11 (8.7)	8 (7.3)
	Cocaine or crack	79 (62.7)	68 (62.4)
**Ever overdosed, n (%)**			
	No	75 (60.5)	70 (64.2)
	Yes	49 (39.5)	39 (35.8)
Percentage of time they share drug equipment^e^, median (IQR)		24.0 (47)	24.5 (48)
Has shared needles, cottons, filters, or cookers in the past 30 days, n (%)		99 (88.4)	87 (87.9)
Has shared needles in the past 30 days, n (%)		78 (70.9)	67 (69.1)
Has shared cottons or filters in the past 30 days, n (%)		76 (71.0)	62 (63.3)
Has shared cookers in the past 30 days, n (%)		86 (81.9)	72 (77.4)
**Frequency of drug use in past 30 days, n (%)**			
	Less than daily	67 (53.2)	63 (57.8)
	Every day	59 (46.8)	46 (42.2)
Years of injection drug use, median (IQR)		5 (13)	7 (13)
**Tested for HCV^f^ before study enrollment, n (%)**			
	No	36 (28.6)	21 (19.3)
	Yes	85 (67.5)	82 (75.2)
	Unsure	5 (4.0)	6 (5.5)
**Result of most recent HCV test, n (%)**			
	Negative	67 (80.7)	62 (79.5)
	Positive	16 (19.3)	16 (20.5)
**Trained to administer Narcan at baseline, n (%)**			
	No	77 (61.1)	62 (56.9)
	Yes	49 (38.9)	47 (43.1)

^a^Column percentages may not add up to 100 because the data are assumed to be missing at random.

^b^GED: general educational development.

^c^HSED: High School Equivalency Diploma.

^d^Statistically significant at α<.05 (unadjusted).

^e^Includes needles, cottons or filters, and cookers.

^f^HCV: hepatitis C virus.

### Randomization Checks

There were no significant differences in demographic variables (gender, race, age, education level, health insurance, employment, income, homelessness, incarceration history, or overdose history) between the intervention and control arms. Those randomized to the control group were significantly more likely to use heroin (92.7% vs 84.3%; *P*=.04) and OxyContin or Oxycodone (50.4% vs 37.6%; *P*=.05) in 30 days before baseline. As the use of these substances may affect the behavioral outcomes assessed in this study, we adjusted for heroin and OxyContin or Oxycodone use in all regression models. There were no significant differences at baseline between the intervention and control participants in the 4 behaviors that Hep-Net targets: the proportion screened for HCV or trained to administer Narcan, nor the frequency of sharing drug equipment or using drugs.

### Main Outcomes

#### Behavioral Goal 1: Receive HCV Follow-Up Testing

Among the 235 study participants, 81 (34.5%) individuals agreed to undergo a rapid HCV test at the time of study enrollment; none of the 32 individuals who self-reported already knowing to be HCV positive agreed to an HCV test at enrollment. Of the 81 individuals, 14 (17%) tested reactive, and all 14 had a blood specimen collected and sent for confirmatory testing.

Of the 203 individuals who did not report testing positive for HCV before study enrollment, 38 (18.7%) received HCV follow-up testing within 6 months of enrollment (Table 2). Individuals in the intervention arm were significantly more likely to undergo HCV follow-up testing than those in the control arm (23.7% vs 14.6%; OR 2.23, 95% CI 1.05-4.74; *P*=.04). This trend persisted at 12 months postenrollment, where 26.9% (25/93) of individuals in the intervention arm and 17.3% (19/110) in the control arm received HCV follow-up testing.

**Table 2:** Proportion who received Hepatitis C virus testing and underwent Narcan training, and the frequency of sharing drug equipment and using drugs at follow-up.

Behavior		Control	Intervention	OR (95% CI)^a^
**Received HCV^b^ follow-up testing within 6 months (n=203), n (%)**				2.23 (1.05-4.74)^c^
	Yes	16 (14.5)	22 (24)	
	No	94 (85.5)	71 (76)	
**Trained to administer Narcan (n=91), n (%)**				2.25 (0.83-6.06)
	Yes	10 (21)	16 (37)	
	No	38 (79)	27 (63)	
**Frequency of sharing drug equipment (n=116)**				0.60 (0.55-0.65)^c^
	Median percentage (IQR)	5 (1-47)	2 (1-24)	
**Frequency of drug use (n=150), n (%)**				0.90 (0.49-1.65)
	Once a week or less	25 (33)	22 (30)	
	More than once a week but not every day	30 (39)	28 (38)	
	Every day	21 (28)	24 (32)	

^a^Adjusted for heroin and OxyContin or Oxycodone use at baseline.

^b^HCV: Hepatitis C virus.

^c^Statistical significance at α<.05.

#### Behavioral Goal 2: Gets Trained to Administer Narcan

Among the 235 study participants, 96 (40.9%) had already been trained to administer Narcan at baseline. Of the 151 individuals who participated in the follow-up survey, 60 were excluded from this analysis because they had been trained before Hep-Net enrollment (n=59) or chose not to answer (n=1). Of the remaining 91 individuals, 26 (28.6%) received training between baseline and follow-up (Table 2). Individuals in the intervention arm were more likely to receive Narcan training than those in the control arm (37.2% vs 20.8%; OR 2.25, 95% CI 0.83-6.06; *P*=.11).

#### Behavioral Goal 3: Reduce Frequency of Sharing Drug Equipment

At baseline, 186/235 (79.1%) reported sharing needles, cottons, filters, or cookers with another person in the 30 days before study enrollment: 145/235 (61.7%) individuals reported sharing injection needles or syringes, 138/235 (58.7%) shared cottons or filters, and 158/235 (67.2%) shared cookers. Thus, the majority of participants shared more than one piece of equipment (98 individuals shared all the 3 pieces, 59 individuals shared 2 pieces, and 29 individuals shared 1 piece). A total of 25/235 (10.6%) individuals reported never sharing any of these 3 pieces of equipment. The median percentage of time participants shared equipment at baseline was 24.0% (IQR 2.0-49.0).

At follow-up, the median percentage of time participants shared equipment dropped to 3.0% overall (IQR 1.0-28.0) among the 116 individuals who provided responses for all 3 pieces of equipment (Table 2). Individuals in the intervention group were significantly less likely to share drug equipment than individuals in the control group (control median, IQR 5, 1-47; intervention median, IQR 2, 1-24). Those in the intervention group shared drug equipment 0.60 times less than those in the control group (OR 0.60, 95% CI 0.55-0.65; *P*<.001).

#### Behavioral Goal 4: Reduce Frequency of Drug Use

Overall, 44.7% (105/235) of individuals reported using drugs every day at baseline, compared with 30.0% (45/150) at follow-up. This reduction in the proportion of individuals using drugs every day was experienced by both intervention and control participants (Table 2), where 32.4% and 27.6% reported using every day, respectively (OR 0.90, 95% CI 0.49-1.65; *P*=.72).

### Readiness to Change

Table 3 shows the distribution of stages of readiness for change at baseline and at follow-up. This table also shows the odds ratios demonstrating the degree to which individuals in the intervention group advanced their stage of readiness in the direction of *maintenance* compared with the control group at follow-up.

**Table 3:** Proportion of individuals in each stage of readiness for risk reduction behaviors at baseline and follow-up and odds ratios comparing the mean values of readiness to change between intervention and control groups at follow-up.

Stage of readiness			HCV^a^ testing intentions	Narcan training intentions	Intentions to use clean works	Intentions to reduce drug use
**Baseline (n=235)** **, n (%)**						
	**Precontemplation and contemplation**					
		Control group^b^	25 (19.8)	34 (27.0)	15 (11.9)	25 (19.8)
		Intervention group^c^	19 (17.4)	24 (22.0)	9 (8.3)	19 (17.4)
		Total	44 (18.7)	58 (24.7)	24 (10.2)	44 (18.7)
	**Preparation**					
		Control group	31 (24.6)	28 (22.2)	13 (10.3)	32 (25.4)
		Intervention group	19 (17.4)	23 (21.1)	17 (15.6)	31 (28.4)
		Total	50 (21.3)	51 (21.7)	30 (12.8)	63 (26.8)
	**Action**					
		Control group	46 (36.5)	23 (18.3)	55 (43.7)	64 (50.8)
		Intervention group	45 (41.3)	20 (18.4)	44 (40.4)	54 (49.5)
		Total	91 (38.8)	43 (18.3)	99 (42.1)	118 (50.2)
	**Maintenance**					
		Control group	24 (19.1)	41 (32.5)	43 (34.1)	5 (4.0)
		Intervention group	26 (23.9)	42 (38.5)	39 (35.8)	5 (4.6)
		Total	50 (21.3)	83 (35.3)	82 (34.9)	10 (4.3)
**Follow-up (n=151)** **, n (%)**						
	**Precontemplation and contemplation**					
		Control group^d^	12 (16)	17 (22)	4 (5)	7 (9.)
		Intervention group^e^	9 (12)	11 (15)	3 (4)	9 (12)
		Total	21 (13.9)	28 (18.5)	7 (4.6)	16 (10.6)
	**Preparation**					
		Control group	4 (5)	10 (13)	3 (4)	11 (14)
		Intervention group	9 (12)	13 (18)	2 (3)	15 (20)
		Total	13 (8.6)	23 (15.2)	5 (3.3)	26 (17.2)
	**Action**					
		Control group	33 (43)	16 (21)	34 (44)	45 (58)
		Intervention group	24 (32)	13 (18)	23 (31)	41 (55)
		Total	57 (37.7)	29 (19.2)	57 (37.7)	86 (57.0)
	**Maintenance**					
		Control group	28 (36)	34 (44)	36 (47)	14 (18)
		Intervention group	32 (43)	37 (50)	46 (62)	9 (12)
		Total	60 (39.7)	71 (47.0)	82 (54.3)	23 (15.2)
OR (95% CI)^f,g^			1.23 (0.67-2.24)	1.28 (0.70-2.34)	1.92 (0.99-3.71)	0.67 (0.36-1.27)

^a^HCV: hepatitis C virus.

^b^n=126.

^c^n=109.

^d^n=77.

^e^n=74

^f^The precontemplation and contemplation stages were combined into a single stage, resulting in a four-category dependent variable (stage of readiness) for each behavior. The probability of reaching the goal (maintenance) was modeled, with control participants being the reference group.

^g^Adjusted for heroin and OxyContin or Oxycodone use at baseline.

#### Behavioral Goal 1: Receive HCV Follow-Up Testing

Overall, the majority of individuals were in the preparation 21.3% (50/235), action 38.7% (91/235), or maintenance 21.3% (50/235) stage of readiness to undergo regular HCV screening. Of the 109 individuals in the intervention group, 3 (2.8%) selected this goal to work on specifically over the next 3 months. At follow-up, a higher proportion of individuals reached the maintenance stage in both groups (60/151, 39.7% overall), with a slightly higher proportion in the intervention group (32/74, 43.2%) than in the control group (28/77, 36.4%). The odds of advancing through the stages of readiness toward the *maintenance* stage were 16% higher in the intervention arm than in the control arm (OR 1.23, 95% CI 0.67-2.24; *P*=.51).

#### Behavioral Goal 2: Get Trained to Administer Narcan

At baseline, 21.7% (51/235) of individuals were in the preparation stage of readiness to undergo Narcan training, 18.3% (43/235) were in the action stage, and 35.3% (83/235) were in the maintenance stage. Of the 109 individuals who received the intervention, 10 (9.2%) chose this goal to work on specifically over the next 3 months. At follow-up, a higher proportion of individuals had reached the maintenance stage overall (71/151, 47.0%), with a slightly higher proportion in the intervention group (37/74, 50.0%) than in the control group (34/77, 44.2%). The odds of advancing through the stages of readiness toward the *maintenance* stage were 28% higher in the intervention arm than in the control arm (OR 1.28, 95% CI 0.70-2.34; *P*=.43).

#### Behavioral Goal 3: Reduce Frequency of Sharing Drug Equipment

At baseline, the highest proportion of individuals were in the action stage (99/235, 42.1%) and the maintenance stage (83/235, 34.9%) for using clean works every time they injected. Among the 109 intervention participants, 17 (15.6%) selected this goal to work on over the next 3 months. The proportion of individuals reaching the maintenance stage at follow-up increased (54.3% overall) among both groups but was higher among intervention participants (62.2%) than among control participants (59/126, 46.8%). The odds of advancing through the stages of readiness toward the *maintenance* stage were 80% higher in the intervention arm than in the control arm (OR 1.92, 95% CI 0.99-3.71; *P*=.05).

#### Behavioral Goal 4: Reduce Frequency of Drug Use

At baseline, the highest proportion of people were in the preparation (63/235, 26.8%) and action (118/235, 50.2%) stages of readiness for the goal of reducing or ceasing drug use. In contrast to the other 3 behavioral goals, the least proportion of individuals were in the maintenance stage (10/235, 4.3% overall). The highest proportion of individuals in the intervention group (72.5%) selected this goal to focus on over the next 3 months. Although the proportion of individuals reaching the maintenance stage at follow-up increased overall to 15.2% (23/151), individuals in the intervention group were less likely to advance along the readiness continuum in the direction of *maintenance* than those in the control group (OR 0.67, 95% CI 0.36-1.27; *P*=.22).

## Discussion

### Principal Findings

The goal of this pilot RCT was to determine whether implementing a computerized risk reduction intervention into existing prevention services at SSPs can improve health-promoting behaviors among people who inject drugs. We found that individuals who received the Hep-Net intervention were significantly more likely to undergo testing for HCV and less likely to share drug equipment. Although not statistically significant, individuals in the intervention arm were also more likely to undergo the Narcan administration training. There was also a trend toward increased readiness to change among intervention participants for these 3 behaviors. However, intervention participants did not appear to reduce the frequency of drug use or increase readiness to reduce drug use more than control participants, despite the fact that the majority of intervention participants selected this as the primary goal to focus on after participation in the baseline survey. These results demonstrate that implementing computer-based risk reduction interventions in SSPs may reduce harms related to sharing of injection equipment and prevent overdose deaths.

No significant difference in reducing the frequency of drug use was observed between the intervention and control participants. Multiple interpretations of this finding are plausible. Hep-Net was a single-session, brief intervention. Reducing substance use is likely to require multipronged strategies, including provision of social, psychological, and physical support. Although Hep-Net in isolation did not affect significant changes in this behavior, it may be useful if integrated into other existing programs to more holistically address the complexity of addiction. Furthermore, almost half of the participants did not return for follow-up. It may be that those who did not return for follow-up disproportionately included individuals who were successful in reducing their substance use and therefore did not return to the needle exchange. Increasing the dosage and comprehensiveness of the intervention may also bolster the effect and allow individuals to reduce drug use and reach their recovery goals. For example, implementing booster sessions that expose clients to additional intervention content may strengthen the effect it has on reducing the frequency of drug use. Understanding how interventions may be enhanced to effectively reduce drug use and improve long-term recovery success among SSP clients is greatly needed.

Although CTIs have become increasingly common for facilitating a wide range of health-promoting behaviors, very few studies have examined the effect of a single CTI on multiple risk behaviors among people who inject drugs in SSP or other community-based settings. With regard to CTIs addressing substance abuse, previous studies have instead primarily focused on HIV/AIDS prevention [34,35], perinatal drug use [36,37], or alcohol use disorders [38,39] or were coupled with therapist-delivered treatments [40,41]. To our knowledge, only one randomized trial has evaluated the efficacy of a CTI for the adoption of safer injection practices among SSP clients. This study found that IV drug users visiting SSPs who received the CTI were using dirty syringes 0.47 times in less than 1 month after the intervention began compared with those who did not receive the CTI; however, the positive effect was short-lived [42]. This study is the first to implement a single CTI into SSPs to address safe injection practices, substance use reduction, hepatitis C testing, and overdose prevention simultaneously. Additional research on CTIs for SSP clients is needed because of its potential to address the challenges of resource limitations facing many affected communities.

The transtheoretical model has been used in the development of various substance abuse interventions and has demonstrated success because it accounts for individuals who are not ready to make behavior change or who do not see their behavior as problematic [30]. However, there is limited research on applying the transtheoretical model to promote healthy behaviors specifically among people who inject drugs, who are engaged in prevention services. Prior studies using transtheoretical model-designed interventions to promote behavior change among people who inject drugs have been restricted to increasing condom use and using bleach to clean drug paraphernalia [43-45]. In this study, we found that most people were in the preparation, action, or maintenance stage for all behaviors at baseline. This heightened readiness may have partially limited our ability to detect the statistical significance in this study.

### Limitations

We demonstrate the effects of a brief, digitally delivered tailored intervention designed to reduce HCV risk behaviors among hard-to-reach individuals who inject drugs. Although our study has much to offer, it is not without limitations. Although delivering the intervention through a well-established SSP that holds strong relationships with many members of the targeted population was a major strength of this study, communities that lack such a developed SSP may fail to reach less-engaged clientele. Generalizability may also be limited because individuals utilizing SSPs may choose to prioritize their health and value safe injection practices more strongly than those not engaged in prevention services, as suggested by the high proportion of individuals in the preparation, action, and maintenance stages of behavior change. For this reason, we implemented a social network–based recruitment strategy to engage difficult-to-reach individuals.

Assessments of drug use behaviors were self-reported measures. However, we anticipate that the computerized approach to data collection maximizes privacy and, in turn, mitigates social desirability bias.

Finally, the primary aim of this pilot study was to assess the feasibility of the social network recruitment approach [29]. As such, the sample size may not be sufficiently powered to detect small effect sizes, particularly for subgroup analyses. Further analysis with improved power is needed to improve our understanding of the effectiveness of computer-tailored risk reduction interventions implemented in SSP settings.

### Conclusions

As the opioid epidemic continues to burden rural communities, expanding the delivery of comprehensive prevention services in resource-poor settings is critically important. To be most effective, prevention services should be patient-centered and provide individual, personalized plans for risk reduction that are responsive to their unique preferences, needs, and motivations. Using fast data-driven decision guidelines, CTIs offer the opportunity to deliver individualized care even in settings facing resource limitations. Implementing CTIs in SSP settings is one approach for engaging highly marginalized and underserved populations of people who inject drugs and facilitate the uptake of safe injection practices, decrease opioid overdose deaths, and reduce the transmission of blood-borne infections. Although such interventions may prevent some devastating consequences of injection drug use, additional efforts are needed to help individuals reduce drug use and overcome the power of addiction.

## Appendix

Multimedia Appendix 1 CONSORT-eHEALTH checklist (V 1.6.1).

## Abbreviations

CTI: computer-tailored intervention
HCV: hepatitis C virus
RCT: randomized controlled trial
SSP: syringe service program
